# Gossiping Robots: A Novel Approach to Enhancing Engagement and Well‐Being in Dementia Care

**DOI:** 10.1002/alz70858_104386

**Published:** 2025-12-25

**Authors:** Arshia A Khan

**Affiliations:** ^1^ University of Minnesota Duluth, Duluth, MN, USA

## Abstract

**Background:**

While often perceived negatively, gossip can act as a powerful social catalyst, reigniting interest in familiar activities among individuals affected by dementia. Studies indicate that 20–40% of community‐dwelling older adults experience symptoms of apathy, while approximately one‐third of individuals aged 85 and older have some form of dementia. Apathy affects 50–70% of people with dementia, contributing to disengagement and social withdrawal. Social robots with conversational capabilities may leverage gossip to stimulate memory recall and encourage participation in meaningful activities.

**Methods:**

We developed a socially interactive robot programmed to deliver positive, familiar, and activity‐based gossip to individuals with dementia. A pilot study was conducted in a memory care facility, where the robot engaged participants, twice a week in conversations designed to rekindle interest in previously abandoned activities. A two‐tailed paired t‐test was performed on pre‐ and post‐intervention assessment scores to evaluate the effectiveness of the approach.

**Results:**

Participants (*n* = 8) showed increased engagement in activities that they had previously discontinued, with improvements in mood and social interaction. Cognitive function, assessed using the MoCA, improved for 6 participants (*p* = 0.0087). Apathy levels, measured by the Apathy Evaluation Scale, decreased for all participants (*p* = 0.00024). Quality of life, assessed through the QoL‐AD, improved for all participants (*p* = 0.0076). Mood, evaluated using the Geriatric Depression Scale (GDS), improved for four participants (*p* = 0.025).

**Conclusion:**

Social robots delivering gossip‐based interactions offer a novel, non‐pharmacological approach to re‐engaging individuals with dementia in meaningful activities. This intervention has the potential to enhance cognitive function, reduce apathy, and improve overall well‐being in dementia care settings.

